# RNAP II CTD tyrosine 1 performs diverse functions in vertebrate cells

**DOI:** 10.7554/eLife.02112

**Published:** 2014-05-08

**Authors:** Jing-Ping Hsin, Wencheng Li, Mainul Hoque, Bin Tian, James L Manley

**Affiliations:** 1 Department of Biological Sciences, Columbia University, New York, United States; 2 Department of Biochemistry and Molecular Biology, Rutgers University New Jersey Medical School, Newark, United States; Howard Hughes Medical Institute, New York University School of Medicine, United States

**Keywords:** RNA polymerase II, CTD, tyrosine, upstream antisense RNA, Rpb1 protein stability, proteasome, chicken, human

## Abstract

The RNA polymerase II largest subunit (Rpb1) contains a unique C-terminal domain (CTD) that plays multiple roles during transcription. The CTD is composed of consensus Y^1^S^2^P^3^T^4^S^5^P^6^S^7^ repeats, in which Ser, Thr and Tyr residues can all be phosphorylated. Here we report analysis of CTD Tyr1 using genetically tractable chicken DT40 cells. Cells expressing an Rpb1 derivative with all Tyr residues mutated to Phe (Rpb1-Y1F) were inviable. Remarkably, Rpb1-Y1F was unstable, degraded to a CTD-less form; however stability, but not cell viability, was fully rescued by restoration of a single C-terminal Tyr (Rpb1-25F+Y). Cytoplasmic and nucleoplasmic Rpb1 was phosphorylated exclusively on Tyr1, and phosphorylation specifically of Tyr1 prevented CTD degradation by the proteasome in vitro. Tyr1 phosphorylation was also detected on chromatin-associated, hyperphosphorylated Rpb1, consistent with a role in transcription. Indeed, we detected accumulation of upstream antisense (ua) RNAs in Rpb1-25F+Y cells, indicating a role for Tyr1 in uaRNA expression.

**DOI:** http://dx.doi.org/10.7554/eLife.02112.001

## eLife digest

When a gene is expressed, the DNA is first transcribed to produce an intermediate molecule called a messenger RNA (mRNA), which is then translated to produce a protein. RNA Polymerase II is an enzyme that makes mRNA molecules in organisms as diverse as plants, animals and yeast.

RNA Polymerase II is a complex made of a number of proteins. The largest protein in this complex includes a ‘carboxy-terminal domain’ that has multiple repeats of seven amino acids one after the other. The first amino acid in each repeat, a tyrosine, is referred to as tyrosine-1. Adding various chemical tags to the amino acids in these repeats co-ordinates the steps involved in the transcription of genes. In yeast, for example, adding a phosphate groups to tyrosine-1 seems to help the polymerase to proceed to make long mRNA molecules. However, it is not known what these chemical tags do in humans or other animals.

Now Hsin et al. (and independently Descostes, Heidemann et al.) have shown that the same phosphate groups on tyrosine-1 perform functions in vertebrates (animals with backbones) that are different to those performed in yeast. These functions include protecting the carboxy-terminal domain from being broken down inside cells, and transcribing the DNA that is upstream of genes.

Hsin et al. replaced tyrosine-1 in RNA Polymerase II from chicken cells with a related amino acid that cannot have phosphate groups added to it. This mutant RNA Polymerase II was unstable and degraded by the molecular machinery in cells that breaks down damaged or unneeded proteins back into amino acids. Hsin et al. also compared the mRNA molecules that are made by the wild-type RNA Polymerase II with those produced by a related mutant. This comparison revealed an unexpected accumulation of RNA molecules that are transcribed in the opposite direction from mRNAs. These RNA molecules, known as ‘upstream antisense RNAs’, have been described only recently. And while the function of these RNAs remains mysterious, the results of Hsin et al. suggest that tyrosine-1 helps to ensure that these RNA molecules are rapidly broken down.

The results of Hsin et al. raise a number of important questions, and foremost among these questions is: how do these newly discovered properties of tyrosine-1 contribute to the control of gene expression in animals? Further work is needed to answer this question.

**DOI:** http://dx.doi.org/10.7554/eLife.02112.002

## Introduction

RNA polymerase II (RNAP II) is a multisubunit enzyme responsible in eukaryotes for transcription of all mRNAs and many non-coding RNAs. Rpb1, the largest subunit, contains a unique C-terminal domain (CTD), composed of up to 52 heptad repeats with the consensus sequence Tyr^1^-Ser^2^-Pro^3^-Thr^4^-Ser^5^-Pro^6^-Ser^7^ (YSPTSPS). The CTD performs multiple functions during transcription (Buratowski, 2009; Munoz et al., 2010; Egloff et al., 2012; Hsin and Manley, 2012; Heidemann et al., 2013), most of which are dependent on phosphorylation of specific CTD residues. For example, Ser5 phosphorylation (Ser5-P) promotes recruitment of capping enzyme (Fabrega et al., 2003) and Ser2-P can be important for 3′ mRNA processing (Kim et al., 2004). Ser7-P and Thr4-P also function in 3′ processing, of snRNAs (Egloff et al., 2007) and histone mRNAs (Hsin et al., 2011), respectively, with the later also functioning in transcription elongation (Hintermair et al., 2012). Tyr1 can be phosphorylated in mammals (by c-Abl; Baskaran et al., 1993) and yeast (Mayer et al., 2012), where it may prevent premature recruitment of termination factors. However, the function(s) of Tyr1 and Tyr1-P in metazoans are unknown.

## Results and discussion

We previously utilized chicken DT40 cells to study properties of the Rpb1 CTD. We showed that an Rpb1 derivative containing a CTD with 26 YSPTSPS repeats (Rpb1-26r) plus the ten C-terminal non-consensus residues, important for stability (Chapman et al., 2005), confers cell viability, while a comparable derivative with all Thr4 residues changed to Val was inviable (Hsin et al., 2011). To investigate the functions of Tyr1, we constructed a plasmid encoding a Flag-tagged Rpb1 derivative, Rpb1-Y1F, identical to Rpb1-26r but with all Tyr1 residues replaced by Phe, and expressed this in Rpb1 conditional knock-out cells (DT40-Rpb1; Hsin et al., 2011). Tyr1 was suggested to be essential for viability in *S. cerevisiae* (West and Corden, 1995), but not in *S. pombe* (Schwer and Shuman, 2011). To determine whether Tyr1 is required for growth in vertebrate cells, DT40-Rpb1 cells were transfected with the Rpb1-Y1F vector, and tetracycline (tet) was added to turn off wild-type Rpb1 expression. Rpb1-Y1F was unable to complement Rpb1, whereas Rpb1-26r fully restored viability (Figure 1—figure supplement 1A).

**Figure 1.**
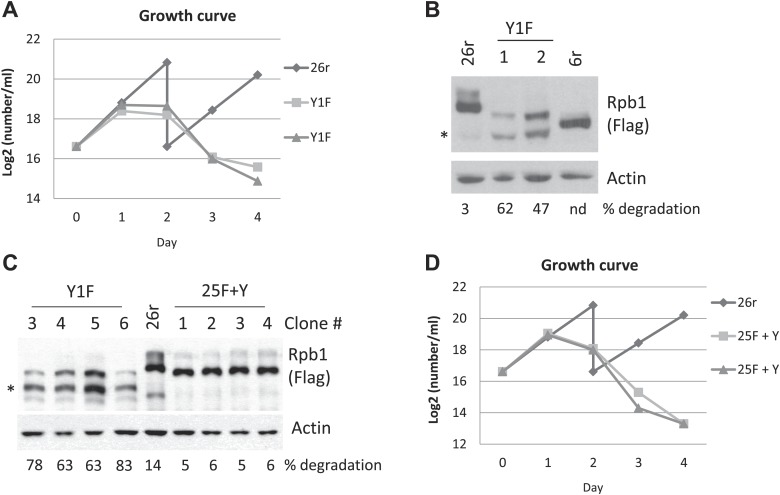
Growth properties of Rpb1 cell lines. (**A**) Cells were cultured in medium containing 1 µg/ml tetracycline (tet). Control cells, 26r, were split on day 2. Average cell counts from two independent experiments were plotted. (**B**) Cells were treated with tet for 24 hr. Whole-cell lysates from 26r, Y1F cells, and cells (6r) expressing an Rpb1 with 6 YSPTSPS repeats were analyzed by western blotting. Flag-tagged Rpb1 proteins were detected using Flag antibody. Partially degraded CTD-less Rpb1 is indicated by an asterisk (*). Full-length and degraded Rpb1 isoforms were quantified using ImageJ, and % degradation is displayed. nd, Degraded Rpb1 not detected. (**C**) Cell lysates from four independent 25F+Y and four independent Y1F cell lines were analyzed as in (**B**). Asterisk indicates partially degraded Rpb1. The lower molecular weight species in the 26r sample is of unknown identity and was not observed reproducibly, but was included in the quantitation. (**D**) Growth curves of two independent 25F+Y cell lines and 26r cells were plotted as in (**A**). **DOI:** http://dx.doi.org/10.7554/eLife.02112.003

We next established cell lines stably expressing Rpb1-Y1F to analyze how the Y1F mutation affects Rpb1 function. Cells expressing Rpb1-Y1F (Y1F) stopped growing around 24 hr in medium containing tet (Figure 1A). To examine whether the inviability of Y1F cells might result from different Rpb1 levels, we analyzed several independent Y1F cell lines by Western blot (WB) with anti-FLAG antibodies (Abs). Rpb1-Y1F levels were indeed significantly reduced compared to Rpb1-26r (Figure 1B). Importantly, accumulation of a lower molecular weight form (indicated by *) was observed in all Y1F cell lines. This corresponds to a derivative likely precisely lacking the CTD, as it migrated slightly more rapidly than an Rpb1 derivative containing six heptads (Figure 1B).

**Figure 1—figure supplement 1.**
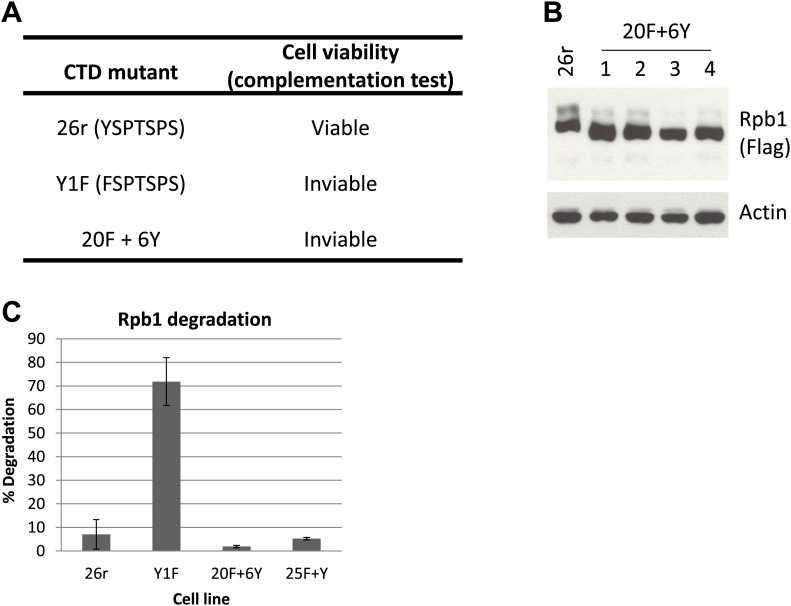
Complementation test and western blotting analysis in cells with Y1F mutations. (**A**) Complementation test was performed as described and results were shown. (**B**) Western blotting analysis of 20F+Y cells. Cells (20F+6Y) expressing an Rpb1 with the last 6 Y1F repeats replaced with normal YSPTSPS repeats were treated with tet for 24 hr. Rpb1 proteins were detected using Flag antibody. (**C**) Whole-cell lysates prepared from cells expressing the indicated Rpb1 derivative and treated with tet for 24 hr were analyzed by western blotting. Western blots were quantified using ImageJ, and % degradation from 3–4 independent replicates is presented. Error bars denote standard deviation. **DOI:** http://dx.doi.org/10.7554/eLife.02112.004

To begin to investigate the basis for Rpb1-Y1F instability, we determined how many Tyr1 residues were necessary to restore stability. We first analyzed an Rpb1-Y1F derivative (20F+6Y) in which the F residues in the C-terminal six heptads were reverted to Y, and found that this derivative was completely stable (Figure 1—figure supplement 1B), although cells expressing Rpb1-20F+6Y remained inviable (Figure 1—figure supplement 1A). Next, we analyzed an Rpb1-Y1F derivative in which only a single F, in the C terminal-most heptad, was changed back to Y (Rpb1-25F+Y). Strikingly, this single Tyr residue was sufficient to prevent Rpb1 degradation, as the truncated isoform, which we denote Rpb1-b, was absent, and Rpb1-25F+Y levels were comparable to Rpb1-26r in multiple 25F+Y cell lines (Figure 1C; quantitation of the amount of degraded Rpb1 observed in multiple experiments is shown in Figure 1—figure supplement 1C). However, despite the restoration of Rpb1 stability, 25F+Y cells remained inviable (Figure 1D).

**Figure 2.**
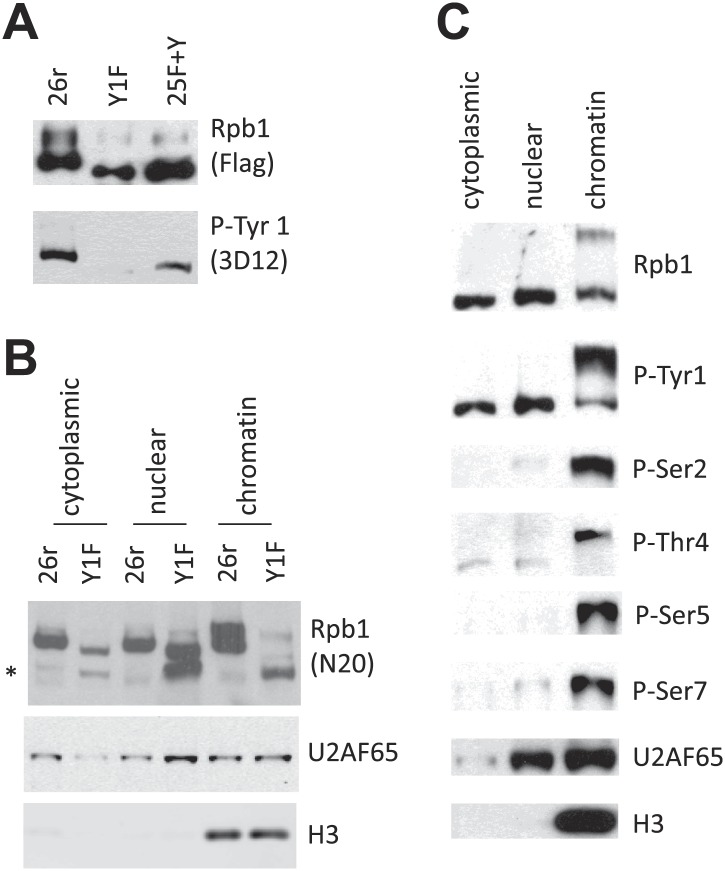
Rpb1 Tyr1 phosphorylation is found in all cell fractions. (**A**) Flag-tagged Rpb1 proteins were immunoprecipitated from cells treated with tet for 24 hr, and analyzed using western blotting. Phosphorylation on Tyr1 (Tyr1-P) was detected by the 3D12 antibody. (**B**) 26r and Y1F cells were treated with tet for 24 hr, subcellular fractionation was performed, and cytoplasmic, nuclear, and chromatin fractions were analyzed by western blotting. U2AF65 (a nuclear protein), and chromatin bound histone H3 protein served as controls for subcellular fractionation. The asterisk (*) indicates partially degraded Rpb1. (**C**) Wild-type DT40 cells were subjected to subcellular fractionation. The localization of Rpb1 phosphorylated on Tyr1, Ser 2, 5 and 7 and Thr4 was determined using antibodies as described in ‘Materials and methods’. **DOI:** http://dx.doi.org/10.7554/eLife.02112.005

We next set out to determine how Tyr1 residues stabilize Rpb1. A first question was whether Rpb1 is indeed Tyr1-phosphorylated in DT40 cells. To address this, we utilized an anti-phospho-Tyr1 Ab (Mayer et al., 2012) to examine Tyr1 phosphorylation (Tyr1-P) of Rpb1-25F+Y and Rpb1-26r by WB; both proteins were indeed Tyr1-phosphorylated (Figure 2A). We next investigated where in cells the Rpb1-b isoform accumulates. We analyzed cytoplasmic, nuclear and chromatin-bound fractions from 26r and Y1F cells by WB with an N-terminal Rpb1 Ab (N20). Rpb1-b (indicated by *) was detected in all three fractions from Y1F cells, but barely or not at all in the 26r fractions (Figure 2B). The relative (and absolute) Rpb1-b levels were lowest in the cytoplasm, while Rpb1-b was essentially the only form on Y1F chromatin. As anticipated, Rpb1-b was not detected in 25F+Y cell fractions (Figure 2—figure supplement 1A). We next determined whether Tyr1-P could also be detected on Rpb1 in all three fractions, in this case using extracts from wild-type DT40 (Figure 2C) and human HEK293 (Figure 2—figure supplement 1B) cells. Robust Tyr1-P was indeed detected in all three fractions in both cell types. Notably, in both cytoplasm and nucleoplasm, Tyr1-P was observed only on hypophosphorylated Rpb1 (the lower band), while it was found primarily on the hyperphosphorylated isoform on chromatin. This suggests both that CTD phosphorylation is limited to Tyr1 in the cytoplasm and nucleoplasm and that Tyr1-P is present on hyperphosphorylated RNAP II found on active genes. We also examined phosphorylation on Ser 2, 5 and 7 and Thr4 (Figure 2C, Figure 2—figure supplement 1B). All these modifications were nearly undetectable in cytoplasmic and nuclear fractions, present almost exclusively on chromatin-associated, hyperphosphorylated Rpb1. Together, our data show that Tyr1, and only Tyr1, is phosphorylated before RNAP II engages in transcription, and support the idea that Tyr1-P functions in stabilizing the CTD when RNAP II is not transcribing, and perhaps also plays a role during transcription. Consistent with this, Tyr1-P was detected on Rpb1 immunoprecipitated by Abs recognizing Ser5-P and Ser2-P (Figure 2—figure supplement 1C).

**Figure 2—figure supplement 1.**
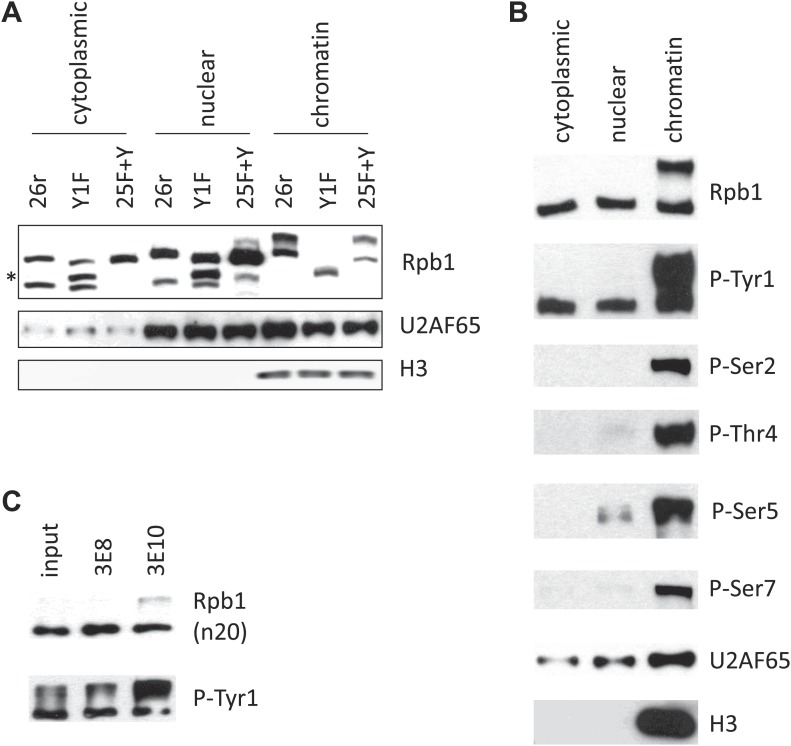
Western blotting analysis. (**A**) Cells were treated with tet for 24 hr, and then subjective to subcellular fractionation. Rpb1 localization was determined by western blotting. Nuclear protein U2AF65, and chromatin-bound histone H3 served as controls for fractionation. Asterisk (*) indicates the degraded Rpb1 fragment. (**B**) Subcellular fractionation assay was performed in HEK293 cells. The localization of Rpb1 phosphorylated on Tyr1, Ser 2, 5, 7, and Thr4 was determined using antibodies as described in ‘Materials and methods’. (**C**) Rpb1 from DT40 cell lysates were immunoprecipitated using antibodies recognizing phosphoserine 5 (3E8) or phosphoserine 2 (3E10). The association of tyrosine phosphorylation with phosphoserine 5 or phosphoserine 2 was determined by western blotting using the 3D12 antibody. **DOI:** http://dx.doi.org/10.7554/eLife.02112.006

**Figure 3.**
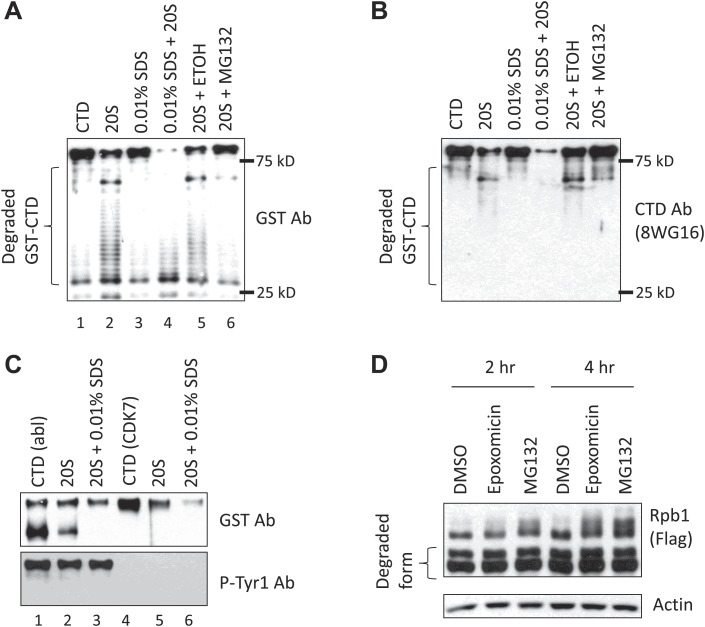
Tyr1 phosphorylation functions in CTD stability. (**A**) *In vitro* 20S proteasome assay. 200 nM purified GST-CTD was incubated with 5 nM bovine 20S proteasome, and the reaction was carried with or without 0.01% SDS. MG132 was used to inhibit the proteasome, and reaction with 2.5% ethanol (ETOH) served as control. Western blotting was performed using antibody against GST, and the CTD (**B**). Position of 25 and 75 kD molecular weight markers are indicated. (**C**) 40 nM GST-CTD phosphorylated by recombinant c-Abl or by purified CDK7 complex was incubated with 2 nM 20S proteasome with or without 0.01% SDS for 1 hr, and reactions were analyzed by western blotting. (**D**) Y1F cells grown in the absence of tet were treated with 50 nM epoxomicin or 5 µM MG132 for indicated time. DMSO treatment served as control. Cell lysates were analyzed by western blotting. Partially degraded Rpb1 is indicated. **DOI:** http://dx.doi.org/10.7554/eLife.02112.007

We next wished to determine how the CTD is degraded, and whether Tyr1-P indeed plays a role. Since one Tyr residue in the final heptad confers stability, it is unlikely that endoproteolytic cleavage occurred between the CTD and the Rpb1 body. One possibility is that the CTD is degraded by the proteasome, which has been shown to associate both with RNAP II and with active genes (Gillette et al., 2004). Additionally, certain naturally unstructured proteins can be degraded by the proteasome in a ubiquitin-independent manner (Sheaff et al., 2000; Tofaris et al., 2001). Since the CTD is considered a structure-less domain (Meinhart et al., 2005), we hypothesized that the CTD is a natural proteasome substrate, and that Tyr1-P prevents its proteasomal degradation. To test this directly, we performed in vitro proteasome assays using a GST-CTD substrate (containing full-length wt CTD) and purified 20S proteasomes, and detected products by WB. Using an anti-GST Ab, the amount of full-length GST-CTD was greatly diminished and a ladder-like pattern of low molecular weight bands was detected, indicating that the proteasome degraded the GST-CTD protein from the C-terminus (Figure 3A, lane 1 and 2). Consistent with this, the low molecular weight products were undetectable by WB using an anti-CTD Ab (8WG16; Figure 3B). Notably, the 20S proteasome used was in a latent status with a closed gate and minimal enzymatic activity (Groll et al., 2000; Forster and Hill, 2003). Thus, GST-CTD, like for example the unstructured protein p21 (Forster and Hill, 2003), was capable of activating the 20S proteasome. Low concentrations of SDS render the proteasome gate disordered, leading to proteasome activation (Groll et al., 2000; Forster and Hill, 2003). Indeed, addition of 0.01% SDS to reaction mixtures increased CTD degradation (Figure 3A, lane 3 and 4). In contrast, the proteasome inhibitor MG132 inhibited degradation (Figure 3A, lane 5 and 6).

**Figure 3—figure supplement 1.**
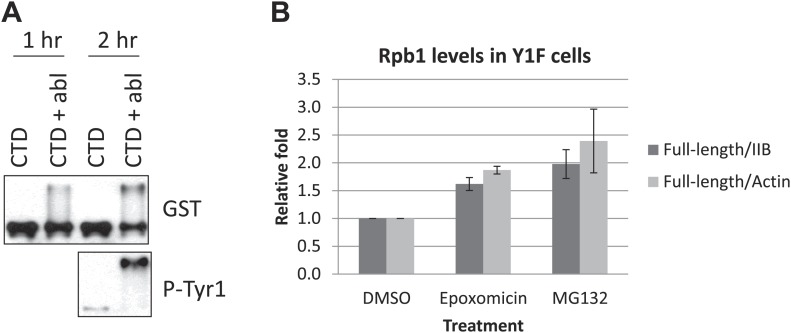
Western blotting analysis and quantification. (**A**) GST-CTD proteins were phosphorylated by Abl tyrosine kinase for indicated time in vitro. Reactions were analyzed by western blotting with indicated antibodies. (**B**) Y1F cells grown in the absence of tet were treated with 50 nM epoxomicin or 5 µM MG132 for 4 hr. Cell lysates were analyzed by western blotting as in Figure 3D. The levels of full-length Rpb1 were quantified using ImageJ, and ratios of full-length Rpb1 to degraded Rpb1 (IIB) and actin were presented. *N* = 3. Error bars display standard deviation. **DOI:** http://dx.doi.org/10.7554/eLife.02112.008

We next asked whether phosphorylation of GST-CTD affects its stability in the proteasome assay. For this, we first used a recombinant c-Abl derivative to phosphorylate GST-CTD. This resulted in conversion of a fraction of the GST-CTD to a low-mobility, Tyr1-P isoform, although the majority remained unphosphorylated (Figure 3C, lane 1, Figure 3—figure supplement 1A), consistent with the processive phosphorylation by c-Abl observed previously (Duyster et al., 1995). We then performed the proteasome assay described above using c-Abl-phosphorylated GST-CTD (Figure 3C). Strikingly, the Tyr1 hyperphosphorylated GST-CTD (top panel, upper band, and lower panel) was resistant to degradation (lane 2), while the remaining unphosphorylated GST-CTD (top panel, bottom band) was degraded. Addition of 0.01% SDS again promoted degradation of unphosphorylated GST-CTD, but the Tyr1-P isoform remained resistant (lane 3). Significantly, GST-CTD phosphorylated by the Ser5/Ser7 kinase CDK7, which converted essentially all of the substrate to the hyperphosphorylated form, was not protected from degradation (lanes 4–6), indicating a specific role of Tyr1-P in preventing proteasomal degradation.

We next investigated whether the proteasome functions in Rpb1-Y1F degradation in vivo. We added the proteasomal inhibitors epoxomicin and MG132 to Y1F cells, and measured intact Rpb1 Y1F levels by WB (Figure 3D). Both inhibitors led to approximately twofold elevated levels of Rpb1-Y1F (see Figure 3—figure supplemental 1B for quantification). Although considerable truncated Rpb1-Y1F remained, this likely reflects incomplete proteasomal inhibition and/or accumulation of the truncated form prior to addition of the inhibitors. In any event, our data provide evidence that Tyr1, and specifically Tyr1-P, prevents proteasomal degradation of the CTD in vitro and in vivo.

**Figure 4.**
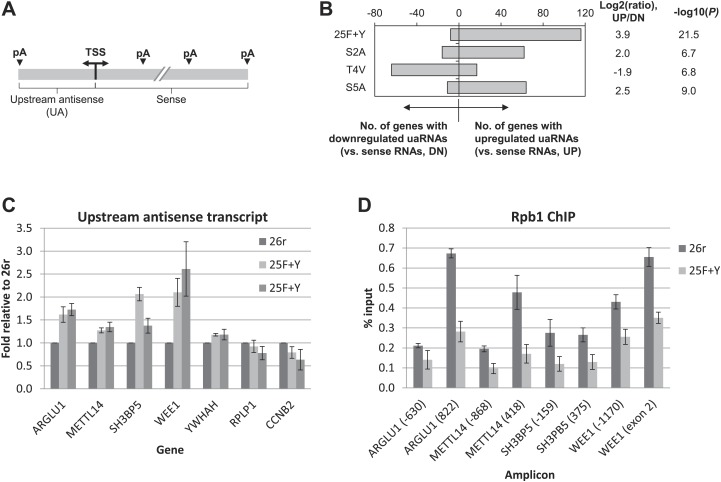
Tyr1 functions in expression of upstream antisense transcripts. (**A**) Schematic of sense RNA and upstream antisense (ua) RNAs analyzed. (**B**) Regulation of uaRNA expression. Cells treated with tet for 24 hr were processed for 3′READS RNA-seq analysis. The number of genes with upregulated (UP) and downregulated (DN) uaRNAs (compared to sense RNA expression) are shown. Their ratio (UP/DN) and p-value (Chi-squared test) are shown. (**C**) RT-qPCR was performed to measure uaRNA levels associated with select genes detected in the RNA-Seq analysis. Fold relative to 26r cells is plotted. *N =* 3. (**D**) Rpb1 levels on sense and antisense genes were determined by ChIP using primers as indicated. The number in parenthesis next to the examined gene indicates the distance between the amplicon and the TSS (minus sign denotes upstream). *N =* 3. Error bars display standard deviation. **DOI:** http://dx.doi.org/10.7554/eLife.02112.009

We next wished to determine the genome-wide impact of the 25F+Y mutation on transcript levels. Using 3′READS (Hoque et al., 2013), a deep sequencing method to quantitate poly(A)+ RNAs, we analyzed 25F+Y and 26r cells, as well as S2A, S5A and T4V cells (all of which, like 25F+Y, are inviable; Hsin et al., 2011; Hsin et al., 2014) for comparison. Cells were treated with tet for 24 hr, and a total of ∼5 million reads mapping to 3′ regions of genes were generated for each cell type (Figure 4—figure supplement 1). Reads were classified into sense RNAs and upstream antisense (ua) RNAs (Figure 4A). uaRNAs were defined as transcripts that did not overlap any known protein-coding genes and used a poly(A) site within 2 kb from the TSS (Figure 4—figure supplement 2). Unexpectedly, the number of genes with upregulated uaRNAs was significantly greater than the number of genes with downregulated uaRNAs, by ∼16-fold (p=10^−21.5^), in 25F+Y cells (Figure 4B). S2A and S5A cells showed similar trends but to much lesser extents, fourfold (p=10^−6.7^) and 5.6-fold (p=10^−9.0^), respectively, while T4V cells in fact showed a trend in the opposite direction (Figure 4B). Using RT-qPCR, we validated several of the uaRNAs (Figure 4C). uaRNAs associated with the *ARGLU1*, *METTL14*, *SH3BP5* and *WEE1* genes were upregulated about twofold in two independent 25F+Y cell lines, consistent with results from RNA-seq (Figure 4—figure supplement 3A). Levels of *RPLP1-* and *CCNB2*-associated uaRNAs were indistinguishable in 26r and 25F+Y cells by both methods.

**Figure 4—figure supplement 1.**
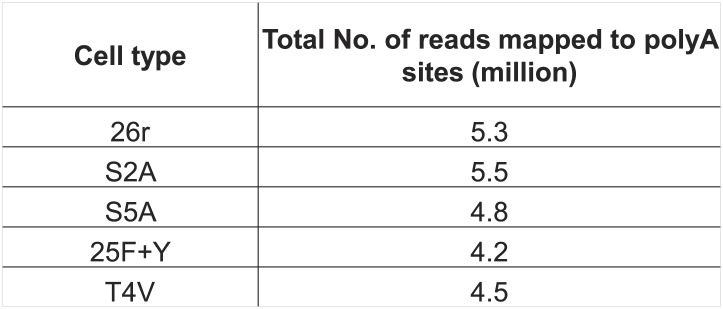
RNA-Seq analysis of Rpb1 cell lines. RNA from cells treated with tet for 24 hr were processed for deep sequencing as described. S2A cells express an Rpb1 derivative with 26 YAPTSPS repeats, whereas S5A cells express an Rpb1 with 28 YSPTAPS repeats. The number of reads mapped to polyA sites for each cell type are shown. **DOI:** http://dx.doi.org/10.7554/eLife.02112.010

**Figure 4—figure supplement 2.**
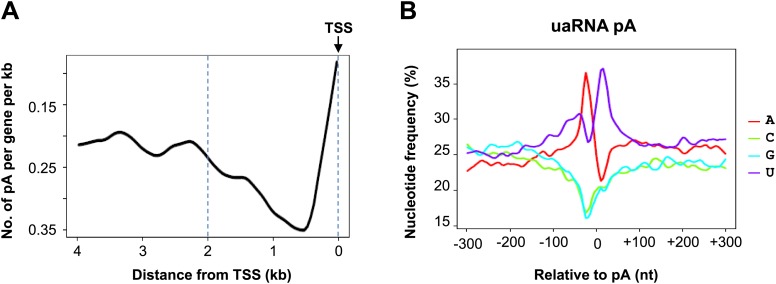
uaRNA polyA site analysis. (**A**) The distribution of uaRNA polyA sites. The number of polyA sites in generated reads was counted and plotted against their distance from TSS. (**B**) The nucleotide profiles of polyA sites in (**A**). **DOI:** http://dx.doi.org/10.7554/eLife.02112.011

**Figure 4—figure supplement 3.**
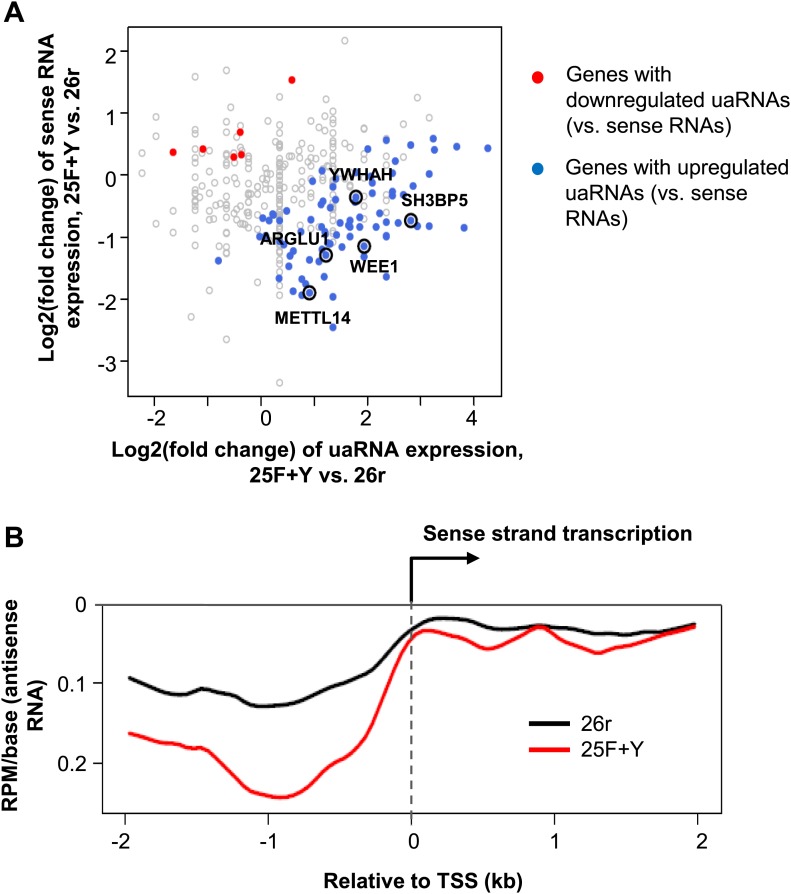
uaRNA expression analysis in 25F+Y and 26r cells. (**A**) Expression difference of uaRNAs vs sense strand RNAs for 25F+Y and 26r cells. Each dot is a gene with uaRNA expression detected. Genes with significant difference in expression of uaRNAs vs sense strand RNAs (p<0.05, Fisher's exact test) were highlighted. The five genes validated in Figure 4C were marked in the plot. (**B**) Expression of antisense poly(A)+ RNA near the transcription start site (TSS) in 26r and 25F+Y cells. Reads per million (RPM) per base for poly(A) sites were shown (y-axis). All genes with upstream antisense (ua) RNAs detected in either 26r or 25F+Y cells were used for plotting. The curves were smoothened by the ‘lowess’ function. **DOI:** http://dx.doi.org/10.7554/eLife.02112.012

**Figure 4—figure supplement 4.**
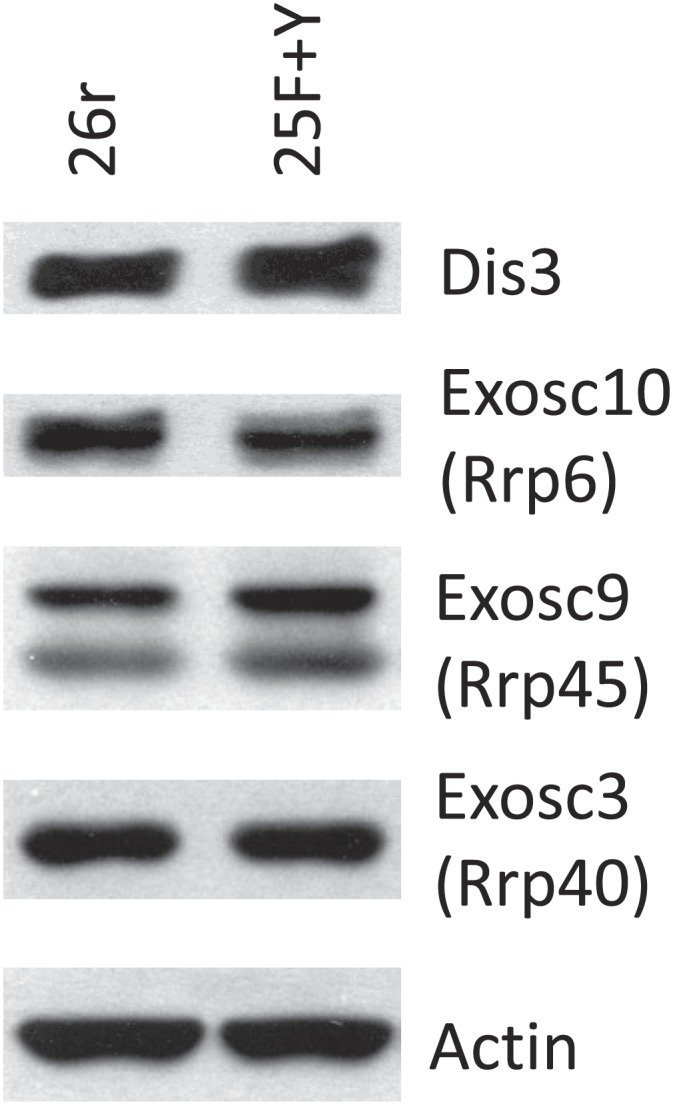
Exosome subunit levels. Cells were treated with tet for 24 hr. The levels of exosome subunits Exosc 3 (Rrp40), Exosc 9 (Rrp45), Exosc 10 (Rrp6), and Dis3 were determined by western blotting. **DOI:** http://dx.doi.org/10.7554/eLife.02112.013

**Figure 4—figure supplement 5.**
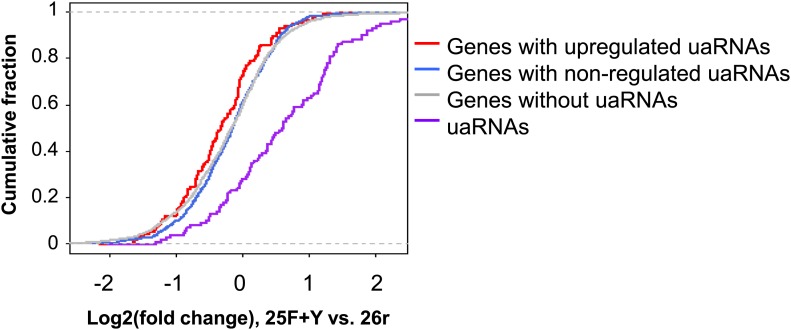
Expression changes for different types of transcripts in 25F+Y cells compared to 26r cells. Values are shown in cumulative distribution function (CDF) curves. Transcript type is indicated in the graph. Expression change was based on log2 ratio of the Read Per Million total PASS reads (RPM) value. We used only genes that had at least 20 reads in two samples combined for this analysis. Expression of uaRNAs (purple line) is significantly upregulated compared to sense transcripts (other lines). Genes with upregulated uaRNAs (red line) tend to be slightly downregulated as compared to genes with non-regulated uaRNAs (blue line) or no detectable uaRNAs (gray line). **DOI:** http://dx.doi.org/10.7554/eLife.02112.014

**Figure 4—figure supplement 6.**
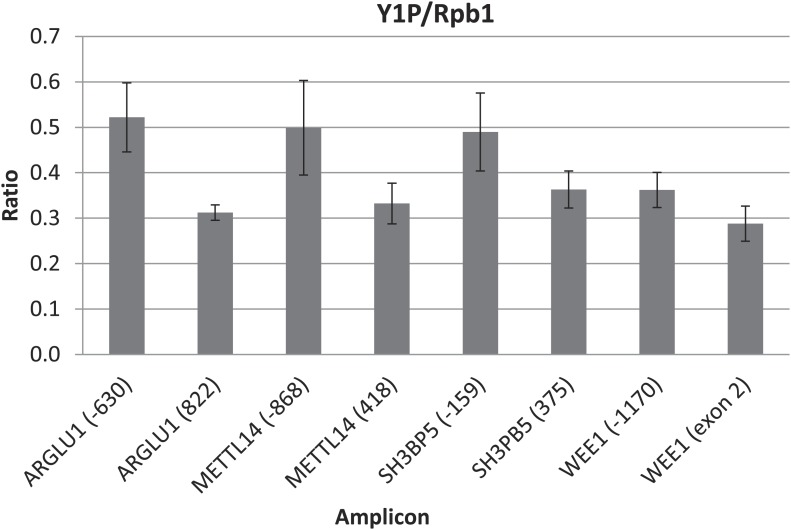
Tyr1-P ChIP analysis. Data from Figure 4D were reanalyzed by normalizing the Tyr1-P signals to Rpb1 levels. *N =* 3. Error bars display standard deviation. **DOI:** http://dx.doi.org/10.7554/eLife.02112.015

We next investigated the mechanism underling the enhanced accumulation of uaRNAs in 25F+Y cells. uaRNAs are low-abundant, usually rapidly degraded by the nuclear exosome (Preker et al., 2008; Seila et al., 2009; Wei et al., 2011; Ntini et al., 2013). However, protein levels of four exosome subunits were comparable in 26r and 25F+Y cells (Figure 4—figure supplement 4), suggesting that the increase in uaRNAs was unlikely due to decreased exosome levels. Also, poly(A) sites of uaRNAs were unchanged (Figure 4—figure supplement 3B), indicating that enhanced accumulation did not reflect altered poly(A) site utilization. Another possibility was that transcription of these transcripts was increased. However, ChIP assays indicated that Rpb1 levels were in fact reduced upstream of the *ARGLU1*, *METTL14*, *SH3BP5* and *WEE1* genes in 25F+Y cells (Figure 4D; see also Figure 4—figure supplement 5). Finally, ChIP analyses showed more Tyr1-P on these upstream genes than on the corresponding downstream sense genes (Figure 4—figure supplement 6). Our results point to a role for Tyr1-P in regulating accumulation of uaRNAs by contributing to their rapid turnover.

In this study, we described two important functions for Tyr1 residues: Protecting the CTD from proteolysis and ensuring turnover of uaRNAs. Both these functions are likely conserved throughout vertebrates, as Descostes et al. (Descostes et al., 2014) report remarkably similar results in human cells. While CTD stabilization requires only a single Tyr1 residue, and Tyr1 phosphorylation, more Tyr1 residues are required for other essential functions. As shown previously (Baskaran et al., 1993, 1999), c-Abl can phosphorylate the CTD in vitro, which stabilizes it from proteasomal degradation. But the role of c-Abl in Tyr1 phosphorylation in vivo is unclear, as c-Abl inhibitors had at most modest effects on Try1-P levels in cells (unpublished data). In any event, our results add additional and unexpected complexity to the multiple functions played by the CTD in controlling RNAP II activity.

## Materials and methods

### Cell culture and cloning

DT40 cells and HEK293 cells were cultured at 37°C with 5% CO_2_ in RPMI1640 medium containing 10% FBS and 1% chicken serum, and in DMEM medium containing 10% FBS, respectively.

Rpb1 CTD derivatives were cloned as previously described (Hsin et al., 2011). Briefly, a fragment of beta-actin promoter and FLAG tag was inserted into pBlueScript containing Neomycin resistance gene. The human *Rpb1* body without the CTD was inserted immediately after the FLAG tag, and various CTD fragments were inserted directly 3′ to the *Rpb1* body.

### Complementation assay and construction of stable cell lines

Procedures for complementation assays and for constructing stable cell clines were followed as previously described (Hsin et al., 2011). Briefly, 10^7^ cells were transfected with linearized DNA, and selected in the presence of appropriate antibiotics. Surviving cell clones were isolated, and the identity of these cells was further confirmed using western blotting.

### Western blotting

Cells lysates, prepared by dissolving washed cells directly in SDS sample buffer, were resolved by SDS-PAGE with indicated percentage of acrylamide. Western blotting was performed using standard protocols. For quantification, western blots were analyzed by ImageJ. Antibodies used in this paper as follows: Flag tag (M2; Sigma, St. Louis, MO), actin (Sigma), phospho CTD Tyr1 (3D12; Active Motif, Carlsbad, CA), U2AF65 (Sigma), histone H3 protein (abcam, Cambridge, MA), phospho CTD Ser2 (3E10; Millipore, Billerica, MA), phospho CTD Ser5 (3E8; Millipore), phospho CTD Ser 7 (4E12, Millipore), Rpb1 CTD (8WG16; abcam), GST tag (Invitrogen, Carlsbad, CA), Rpb1 (N20; Santa Cruz, Santa Cruz, CA), Exosc10 (Rrp6) (Novus, Littleton, CO), Exosc9 (Rrp45) (Novus), Exosc3 (Rrp40) (Novus), and Dis3 (Novus).

### Subcellular fractionation

Subcellular fractionation was performed using a modified protocol as described (Mapendano et al., 2010). Briefly, cells (1 ∼ 2 × 10^7^) were harvested, washed in PBS, and resuspended in 0.5 ml of RSB100 (50 mM Tris–HCl PH 7.4, 100 mM NaCl) containing 40 µg/ml digitonin. Cell extracts were incubated on ice for 5 min. The cytoplasmic fraction was separated from nuclear fraction by centrifugation (2000×*g*, 5 min). The pellets were resuspended in 0.5 ml of RSB100 containing 0.5% Triton X-100, and the reactions were incubated on ice for 5 min. Separation of soluble nuclear proteins from insoluble chromatin bound proteins was carried by centrifugation (2000×*g*, 5 min). The pellets containing chromatin bound proteins were resuspended in 0.5 ml of RSB100 (0.5% Triton-X100), and sonicated briefly.

### In vitro proteasome assay

In vitro proteasome assays were performed as described (Asher et al., 2005) with the following modifications. Briefly, GST-CTD or GST-CTD phosphorylated by abl tyrosine kinase was incubated with 2–10 nM bovine 20S proteasome (UBPBio, Aurora, CO) in a buffer (50 mM Tris–HCl PH 7.4, 100 mM NaCl, 0.5 mM EDTA) at 37°C for 1 hr. Reactions were stopped by adding equal volume of 2X SDS PAGE sampling buffer.

### In vitro phosphorylation of GST-CTD

CDK7 complexes were expressed in insect cells, and purified using Ni-NTA agarose (QIAGEN, Valencia, CA) as described (Larochelle et al., 2006). GST-CTD was expressed in *E. coli* and purified using glutathione Sepharose 4B (GE Healthcare). Phosphorylation of GST-CTD by CDK7 complexes was carried out at 30°C for 1 hr in a kinase buffer (25 mM Hepes PH 7.5, 10 mM MgCl_2_, 150 mM NaCl, 1 mM ATP). GST-CTD phosphorylation by recombinant c-Abl kinase (NEB, Ipswich, MA) was performed as described in manual. Briefly, 500 nM GST-CTD was incubated with 25 U c-Abl at 30°C for 2 hr. Phosphorylated GST-CTD was purified using glutathione Sepharose 4B (GE Healthcare, Pittsburgh, PA).

### RT-qPCR

RNA was extracted using Trizol (Invitrogen), and further treated with DNase I. Reverse transcription and qPCR analysis were performed as previously described (Hsin et al., 2011). Primer sequences are listed in Supplementary file 1.

### Immunoprecipitation

About 2 × 10^7^ cells were collected, and washed with PBS. Then, 1 ml cold RIPA (150 mM NaCl, 1 mM EDTA, 50 mM Tris–HCl pH 7.4, 0.5% NP-40, 0.25% sodium deoxycholate) buffer containing 1X PhosSTOP (Roche, Madison, WI), and 1X protease inhibitors (1.4 µg/ml Pepstatin A, 0.35 µg/ml Leupeptin, and 1.7 µg/ml Aprotinin). After brief sonication, debris was centrifuged at 12,000×*g*, 4°C, for 10 min, and the supernatant was removed to a new tube. 50 µl of the lysate were kept for input control, and the rest of the extract was incubated with 20 µl of pre-washed protein G Sepharose and 1–4 µg of antibody. Samples were rotated at 4°C for 1–2 hr, and beads were washed with cold RIPA buffer for 3 min three times, and then were resuspended in 100 ul of 1X SDS sample buffer for western blotting.

### Chromatin immunoprecipitation (ChIP)

Cells were grown to 70% confluence (∼2 × 10^6^/ml), cross-linked with 1% formaldehyde for 10 min, and processed for ChIP as previously described (Hsin et al., 2011). ChIP was performed using antibody against Flag tag (M2; Sigma), phospho CTD Tyr1 (3D12; Active Motif). Primers sequences are listed in Supplementary file 1.

### Deep sequencing and data analysis

Total RNA was extracted from cells treated with tet for 24 hr. RNA was further processed, and analyzed by 3′READs, a deep sequencing method to analyze poly(A)+ RNAs using 3′ end regions, as described (Hoque et al., 2013) with some modifications for RNA fragmentation. Briefly, poly(A) RNA was selected using oligo d(T)25 magenetic beads (NEB), followed by fragmentation of RNA on-bead using RNaseIII (NEB). We generated two libraries for each cell type (biological replicates), and ∼4 million reads for each library. cDNA insert size range corresponded to RNA fragments of ∼100–200 nt. The reads were mapped to the chicken genome (version galGal4), and those with at least two non-genomic As at the 3′ end were considered as poly(A) site-supporting (PASS) reads. PASS reads were assigned to protein coding genes defined by Refseq. The 3′ end of each gene was extended by 4 kb if there was no gene on the same strand within this region. The PASS reads mapped to genic regions are called sense strand reads. Those mapped to the 2 kb upstream region of transcription start site (TSS) on the antisense strand were called upstream antisense (ua) RNA reads. We also required that uaRNA reads could not be assigned to any other annotated genes as sense strand reads. To examine expression change of uaRNAs vs sense RNAs, we grouped all uaRNA reads and compared them to all sense reads of a gene between two samples, for example, 25F+Y vs 26r. Genes with p-value <0.05 (Fisher′s exact test) were selected.

## Additional files

Supplementary file 1. List of primer sequences. **DOI:** http://dx.doi.org/10.7554/eLife.02112.016
