# Supplementary material for: RNAP II CTD tyrosine 1 performs diverse functions in vertebrate cells
Source: eLife. 2014 May 8;3:e02112. doi: 10.7554/eLife.02112 (PMC4042873; doi:10.7554/eLife.02112)
Supplement: Supplementary file 1. — List of primer sequences. DOI: http://dx.doi.org/10.7554/eLife.02112.016 [file elife02112s001.docx]

**Supplemental Table 1.** List of primer sequences.

| Amplicon | Primer | Sequence |
| --- | --- | --- |
| ARGLU1 | ARGLU1_5'_F/R | TGCAGTGAGACGTGACATCT |
|  | ARGLU1_5'_F/R | AGTGGCTGTGTCCTCGTTAA |
| METTL14 | METTL14_p_F1 | AGCCACCGTCAATAACAGGA |
|  | METTL14_p_R1 | TAACGGCGCTGAGATTGAGA |
| SH3BP5 | SH3BP5_p_F/R | ACCACCCTCAAATCAGACCA |
|  | SH3BP5_p_F/R | GGAGGAAGGGAGAGCGAAAT |
| WEE1 | wee1_p_F2/R2 | GCAATTTGTCGGGGCTTTTGT |
|  | wee1_p_F2/R2 | TTACTGCAGCACACCGAAAC |
| YWHAH | YWHAH_5'_F/R | CTGACAGTGTTTTCGTGGACA |
|  | YWHAH_5'_F/R | TAAAACCACTCACTCGCCCA |
| RPLP1 | rplp1_p_F/R | GCTCTAGCTCTCCGTGCTTC |
|  | rplp1_p_F/R | TAAGCAGATCCCACGCTGAT |
| CCNB2 | ccnb2_p_F/R | GGTACCGCTTCCTCAGCTC |
|  | ccnb2_p_F/R | CTCTTTTTCGTCCCCACCTT |
| ARGLU1 (-630) | ARGLU1_5'_F | TGCAGTGAGACGTGACATCT |
|  | ARGLU1_5'_R | AGTGGCTGTGTCCTCGTTAA |
| ARGLU1 (822) | ARGLU1_F1 | CTTCCTCCCCTAAGCTGGAG |
|  | ARGLU1_R1 | TTTCGGGAAGGGAAGGATGG |
| METTL14 (-868) | METTL14_p_F | AATGTCCACCCTCAAAACGC |
|  | METTL14_p_R | ACAGAGATCGTTCCAGCAAG |
| METTL14 (418) | METTL14_F3 | GTGAGCTTGTTGGGTTCTCG |
|  | METTL14_R3 | ACAAAACAGAAACAGCAGAGCA |
| SH3BP5 (-159) | SH3PB5_p_F1 | GCAGGGAGGAACGACAAAAG |
|  | SH3PB5_p_R1 | GGAGGAAGGGAGAGCGAAAT |
| SH3BP5 (375) | SH3BP5_F1 | GAAATGCGGGGATGGGAAC |
|  | SH3BP5_R1 | CTGCCATTACAGCTCCTTCT |
| WEE1 (-1170) | wee1_5'_F | GCCATCGTGCTCAACCAAAT |
|  | wee1_5'_R | CTCATCTCCAGGAGCAGAGC |
| WEE1 (exon 2) | wee1_2F | CTGGAGGAGGAGGAGGAGAT |
|  | wee1_2R | GAAGCCCTCCTCCTCCCA |
